# Natural course of perianal abscess in infants: a real-world study

**DOI:** 10.1038/s41598-023-45751-7

**Published:** 2023-10-27

**Authors:** Wanbin Yin, Yansen Li, Jingfeng Zhang, Yang Jiao, Wenju Pei, Xiangjun Xu, Mingfeng Fan, Juan Xu, Yue Zhou, Shuai Wang, Yanhua Wei

**Affiliations:** 1 Department of Anorectal Surgery, Affiliated Hospital of Jining Medical University, 129 Hehua Road, Jining, 272067 China; 2 Department of Infectious Diseases, Affiliated Hospital of Jining Medical University, 129 Hehua Road, Jining, 272067 China

**Keywords:** Diseases, Health care, Medical research

## Abstract

Natural course of perianal abscess (PA) in infancy remains obscure. This study aimed to investigate the natural course of infants with PA after conservative treatment. A retrospective cohort study was conducted in infants with PA who were treated conservatively (due to the parents’ refusal of surgery), for more than 2 months between 2014 and 2020 at a single tertiary center. 153 patients (149 boys and 4 girls) were identified. The median follow-up was 5.3 years (range 3–8.2 years). Initially, 119 patients (77.8%) were completely cured by conservative treatment, and 34 (22.2%) failed. Among the 34 patients, 23 continued conservative treatment (20 cure, 3 fistula formation) and 11 underwent surgery. After conservative treatment, the rate of fistula formation, abscess recurrence, and new-onset abscess were 15.0%, 4.6%, and 6.5%, respectively. Overall, 139 patients (90.8%) were cured conservatively without surgery, and 11 (7.2%) underwent surgical management. In addition, 3 (2.0%) patients developed fistula-in-ano (under observation). PA in infants may be a time-limited and self-limited condition. Conservative management should be the first choice of treatment in most cases. Longer periods of conservative treatment may achieve better clinical outcomes in selected cases. There will be a percentage of patients (about 10%) that would require surgical treatment.

## Introduction

Although perianal abscess (PA) is a common purulent disease, PA in infants is poorly understood. The clinical features and treatment of PA are different in infants compared to adults. Unfortunately, PA in infants has seldom been focused on until now.

The optimal management of PA in infants has not yet been established. Traditionally, incision and drainage (ID) is the most common treatment for PA in infants^1–3^. In addition, some studies have suggested that when a fistula is identified and laid open at the time of primary drainage, the rate of recurrence or fistula formation may be significantly reduced^4–8^. However, it is noteworthy that some studies have shown that PA in infants is a time-limited and self-limited disorder since 1998^9,11^. They found that nonoperative management of PA in infants appears to be safe and effective. In contrast, other studies presented the opposite view: PA cannot be cured by a period of conservation^12^, and conservative treatment of PA is permanently efficient in only a minority of children^13^. Thus, conservative management of PA in infants remains controversial.

Although conservative treatment has been recommended during the last two decades, it has not yet been widely accepted for far. It is worthy to note that a large number of surgeons still favor incision and drainage with or without primary fistulotomy. In addition, literature on conservative treatment for PA in infants is currently scarce. What’s worse, most of the studies were single-center retrospective studies with a relatively small sample size. In light of the limitations of the previous studies, additional study is required to ascertain the reproducibility.

In our center, the nonoperative management of PA in infants and children was not routinely performed. Our previous study demonstrated that PA in neonates can be treated safely and effectively by ID or ID with fistulotomy^14^. Nevertheless, we indeed observed that 3 of the 4 patients who experienced spontaneous drainage were cured without surgery (due to parental refusal), as mentioned in the discussion. Despite surgical treatment being recommended for the patients, many parents refused surgery because their infants were too young. Therefore, we were particularly interested to know if the patients are treated without surgery, how many patients would be cured without surgery, and how many patients would fail conservative treatment. These questions may shed light on the natural course of PA in infants. Infants with PA would be cured largely without surgery if PA in infants is a time-limited disorder.

The present real-world study aimed to investigate the natural course of infants with PA by studying the clinical outcomes of conservative treatment for PA in infants whose parents refused surgery.

## Materials and methods

### Settings and patients

A retrospective review was carried out for all consecutive infants (< 1 year) with PA in the outpatient clinic of the Affiliated Hospital of Jining Medical University (a tertiary center) between September 2014 and December 2020. The study subjects were infants with PA who didn’t undergo surgery due to the refusal of their parents. Conservative treatment was not uniform, which consisted of sitz baths with traditional Chinese medicine, hygiene, erythromycin ointment, and mupirocin ointment, et al. The natural course of PA in infants may be affected if the duration of conservative treatment is insufficient, so patients who underwent surgical treatment within 2 months of conservative treatment were excluded.

### Diagnostic criteria

The diagnostic criteria of PA, recurrence of PA, and new-onset PA were based on our previous study^14^. FIA was diagnosed by the presence of a hole with or without pus drainage at the site of the anus, persisting more than 2 months after conservative treatment.

### Inclusion and exclusion criteria

The inclusion criteria were infants with PA (< 1 year) attending outpatient clinics of our center whose parents refused surgery. Exclusion criteria were patients who had undergone surgical treatment at other centers before the clinic visit, patients who underwent surgery shortly after the clinic visit or within 2 months of the clinic visit in our or other hospitals, or patients lost to follow-up.

### Follow-up

Follow-up data were mainly obtained by telephone interview, supplemented by outpatient review.

### Data collection

Collected data included clinical and demographic characteristics, whether or not surgery was performed, fistula formation, recurrence of the abscess, new-onset abscess, and length of follow-up.

### Statistical analysis

Continuous variables were converted into categorical variables, which were summarized as frequencies (%). This study did not involve any statistical analysis.

### Ethical approval

This study was approved by the Affiliated Hospital of Jining Medical University Institutional Review Board. All methods were performed in accordance with the relevant guidelines and regulations. Due to the observational and retrospective nature of the study, informed consent was waived off from the Affiliated Hospital of Jining Medical University Institutional Review Board.

STROBE guidelines were followed for reporting.

## Results

**Figure 1:**
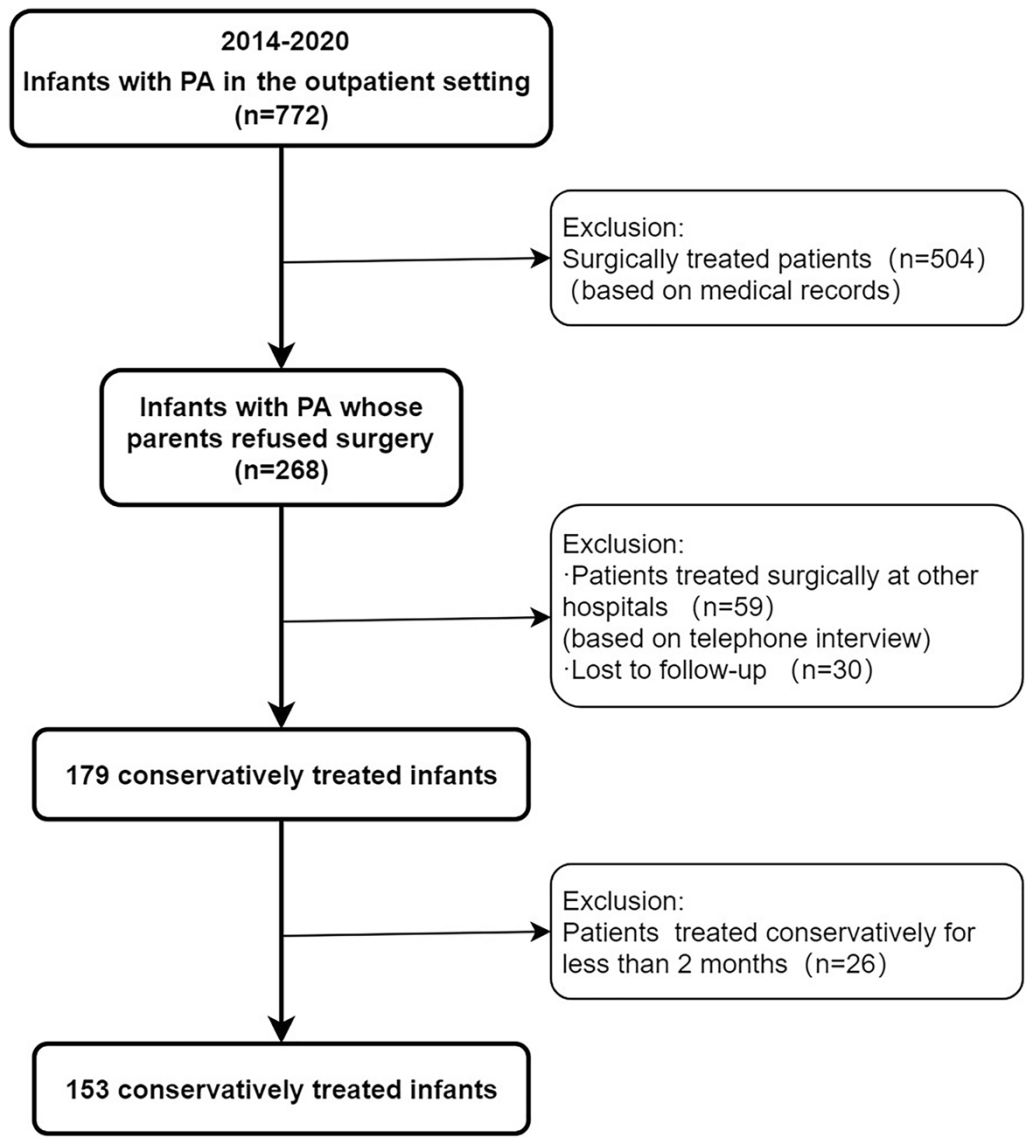
The flowchart of patient inclusion. *PA* perianal abscess.

Overall, 772 infants with PA (< 1 year) were identified in the outpatient setting from August 2014 to December 2020. Among the 268 infants whose parents refused surgery, 30 (11.2%) were lost to follow-up. According to the inclusion and exclusion criteria, a total of 153 eligible infants were included in the final analysis. The study screening flow chart is shown in Fig. 1.

Clinical and demographic characteristics of infants with PA at their first visit are summarized in Table 1. The median follow-up time was 5.3 years with a maximum of 8.2 years. Among the 153 patients included, 86 (56.2%) had abscesses smaller than 1 cm, and 67 (43.8%) larger than 1 cm.

**Table 1:** Clinical and demographic characteristics of infants with PA at first visit.

Variables	n (%)
Gender	
Male, n (%)	149 (97.4)
Female n (%)	4 (2.6)
Age of onset, m	
0–3	120 (78.4)
3–6	17 (11.1)
6–9	12 (7.8)
9–12	4 (2.6)
Number of the abscess, n (%)	
1	144 (94.1)
2	9 (5.9)
Locations of the abscesses	
Left, n (%)	61 (39.9)
Right, n (%)	81 (52.9)
Left and right, n (%)	9 (5.9)
Front, n (%)	2 (1.3)
Size of the abscesses	
< 1 cm	86 (56.2)
≥ 1 cm	67 (43.8)
Fever	
Yes	7 (4.6)
No	146 (95.4)

*PA* perianal abscess, *m* month.

**Figure 2:**
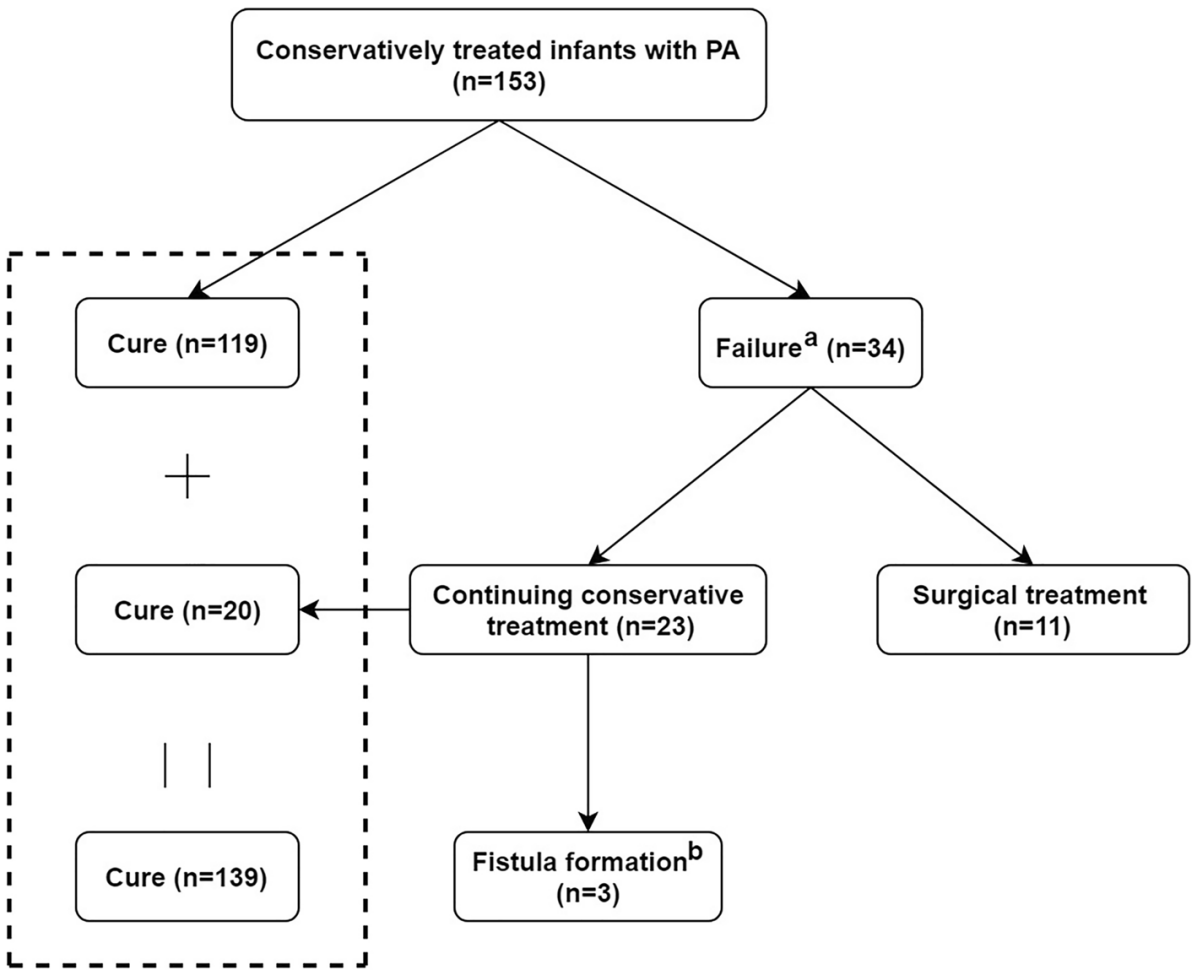
Outcomes of conservative treatment for PA in infants. ^a^Failure includes fistula formation, abscess recurrence, or new-onset abscess. ^b^These three patients with infrequent symptoms are under observation without surgery. *PA* perianal abscess.

Clinical outcomes of conservative management for infants with PA are shown in Fig. 2. There were 34 patients who failed initial conservative treatment. Among the 34 patients, 11 underwent surgical treatment (Table 2), and 23 continued conservative treatment (Table 3). Surprisingly, among the 23 patients, 20 were cured at last after continuation of conservative treatment. Overall (based on Tables 2, 3), 23 patients developed fistula formation, 7 developed abscess recurrence, and 10 developed new-onset abscess after conservative treatment. Therefore, the rate of fistula formation, abscess recurrence, and new-onset abscess were 15.0%, 4.6%, and 6.5%, respectively.

**Table 2:** Characteristics and outcomes of patients who underwent surgery after failure of conservative treatment.

Case	Fistula formation	Abscess recurrence	New-onset abscess	Time to surgery (m)^a^	Treatment	Follow-up time (m)
1	No	No	Yes	2	IDF	42
2	Yes	No	No	2.5	Fistulotomy	68
3	Yes	No	No	3.5	Fistulotomy	33
4	Yes	No	No	4	Fistulotomy	69
5	No	Yes	No	5	IDF	71
6	Yes	No	Yes	9	Fistulotomy	40
7	Yes	No	Yes^b^	11	Fistulotomy	54
8	Yes	No	Yes^c^	18	Fistulotomy	72
9	No	Yes	No	5	ID	44
10	No	Yes	No	3	ID	70
11	No	Yes	No	14	ID	27

*ID* incision and drainage, *IDF* incision and drainage with primary fistulotomy, *m* month.

^a^Time from first onset of symptom to surgery.

^b^There were 4 new-onset abscesses, all of which developed into fistula-in-ano.

^c^There were 3 new-onset abscesses, all of which developed into fistula-in-ano.

**Table 3:** Characteristics and outcomes of patients who continued conservative treatment after failure of conservative treatment.

Case	Fistula formation	Abscess recurrence	New-onset abscess	Outcome	Time to cure (m)	Follow-up time (m)
1	No	No	Yes	Cure	2	36
2	No	No	Yes	Cure	2	65
3	Yes	No	No	Cure	3	41
4	Yes	No	Yes	Cure	3	41
5	No	Yes	No	Cure	3	57
6	No	No	Yes	Cure	4	22
7	Yes	No	No	Cure	5	48
8	Yes	No	No	Cure	5	86
9	Yes	No	Yes	Cure	6	34
10	Yes	No	No	Cure	6	77
11	Yes	No	No	Cure	6	64
12	Yes	No	No	Cure	6	38
13	Yes	No	No	Cure	6	32
14	No	Yes	No	Cure	6.5	34
15	Yes	No	Yes	Cure	7	21
16	Yes	No	No	Cure	7	26
17	Yes	No	No	Cure	10	38
18	No	Yes	No	Cure	10	25
19	Yes	No	No	Cure	12	51
20	Yes	No	No	Cure	12	78
21	Yes	No	No	Fistula formation^a^		18
22	Yes	No	No	Fistula formation^a^		77
23	Yes	No	No	Fistula formation^a^		28

*m* month.

^a^These three patients with infrequent symptoms are under observation without surgery.

Amazingly, the overall cure rate of conservative treatment was as high as 90.3% (139/153). The proportion of patients who received surgical treatment was 7.2% (11/153). 3 (2.0%, 3/153) patients developed fistula-in-ano and are currently under observation due to the infrequent symptoms.

## Discussion

This retrospective study investigated the natural course of PA in infants. The results demonstrated that the overall cure rate of conservative treatment for PA in infants was as high as 90.3% (139/153). Only a minority of patients (7.2%: 11/153) underwent surgery. After conservative treatment, the rate of fistula formation, abscess recurrence, and new-onset abscess were 15.0%, 4.6%, and 6.5%, respectively. These findings supported that PA in infants may be a time-limited and self-limited disease. Conservative management of PA in infants is promising, and early surgical treatment should be avoided as much as possible.

Surgical management was routinely performed for infants with PA at our center before 2023. The subjects in this study were infants with PA who were treated conservatively because their parents refused surgery. To the best of our knowledge, no previous studies have investigated the clinical outcomes of infants with PA whose parents refused surgery.

There were new findings in our study. Our results also showed that longer periods of conservative treatment may achieve better clinical outcomes in selected cases. Among the 34 patients who failed initial conservative treatment, 11 underwent surgical treatment, and 23 continued conservative treatment. Surprisingly, among the 23 patients, 20 were cured at last and 3 patients developed fistula-in-ano (under observation due to the infrequent symptoms). Therefore, we speculate that conservative treatment can be continued for a relatively longer period for those who failed initial conservative treatment in selected cases.

These were particularly surprising and encouraging findings. We believed that there was a possibility of a self-resolution of PA before we conducted this work. However, we did not expect that the overall cure rate of conservative treatment for PA would be so high. There is no doubt that conservative treatment should be the first choice for PA in infants in most cases. Nonetheless, it should be recognized that conservative treatment cannot guarantee a successful cure for all patients. Operative management should be reserved for patients who failed conservative treatment.

Our previous study suggested that PA in neonates can be treated safely and effectively by ID or ID with fistulotomy^14^. These results appear to contradict those of the present study. However, it needs to be stated that our previous report was based on the fact that surgical management was routinely performed for infants and children with PA at our center. As mentioned in the discussion, among the 4 patients with spontaneous drainage, 3 were cured spontaneously without surgery. The importance of conservative treatment for PA in infants has been recognized since then. Here, it is important to emphasize that although ID or ID with fistulotomy of PA is safe and effective in infants, early surgical management should be avoided because of the high cure rate of conservative treatment.

Since the late 1990s, some studies have shown that PA in infancy is a time-limited disorder and that conservative treatment is safe and effective for most infants with PA. In 1998, Watanabe et al. demonstrated that PA in infancy is likely to be a time-limited disorder that occurs mainly in infants and almost always spontaneously resolves within the first year of life^9^. They analyzed the natural course of PA in 97 children. Their results showed that more than 90% of the infants with PA were cured without fistulotomy or fistulectomy. These results are highly consistent with our study. Later, in 2000, Rosen et al. performed a prospective conservative approach to PA in 18 male infants^10^. In their study, PA was to be drained only if the infant was very uncomfortable or febrile. Once a fistula developed, they continued observation until the fistula healed. 4 patients underwent ID for discomfort or fever. Of the remaining 14 infants whose abscesses drained spontaneously, 1 was cured, and 13 developed fistula-in-ano. Of note, all 13 patients with fistula formation had complete healing without operation. Based on these results, they suggested that PA in infants is a self-limited condition rarely requiring surgical drainage. Their results are largely consistent with our observations. Furthermore, the nonoperative management of PA in infants has been supported by other studies over the last two decades^11,15–27^.

However, some studies have reported different points of view. Oh et al. recommended surgical management for PA in infants in 2001^12^. In their retrospective study, 18 patients with PA displayed an onset of symptoms in infancy and a duration of symptoms longer than 12 months, all of whom, failing nonoperative management, underwent fistulotomy. However, there was one main limitation in this study: not all of the patients treated conservatively were enrolled. As mentioned in their discussion, the patients who were cured with nonoperative management usually did not visit the hospital when their lesions disappeared. In other words, their study only included patients who failed conservative treatment. Thus, this study suffered from an obvious selection bias. In contrast, our study enrolled all conservatively treated patients, which made our results more credible. Furthermore, in 2020, Boenicke et al. suggested that conservative treatment of PA is permanently efficient in only a minority of children^13^. The main limitation of this study was that the conservative treatment duration was too short (only 2 weeks) to demonstrate efficacy. PA in infants may spontaneously resolves within the first year of life. If the conservative treatment had lasted longer, the patients may have had better outcomes. In the present study, to better understand the natural course of PA in infants, only patients treated conservatively for more than 2 months were included. We suggest that infants with PA receive sufficient conservative treatment before resorting to surgical intervention.

Additionally, our study showed that after conservative treatment 10 patients developed a new-onset abscess and the rate of new-onset abscess was 6.5% (10/153). Surprisingly, this was exactly the same as in our previous study^14^ which showed that 6.5% (9/138) of neonatal patients developed new abscesses after surgery. New-onset abscess is unique to children with PA compared to adults.

There are several strengths of the present study. First, the natural course of PA in infants, rarely reported in the literature, is an extremely interesting and valuable topic. Our results shed some light on this important question. Second, this was a real-world study that reflected real clinical conditions. Third, the sample size of our study was large (153 infants). Fourth, the follow-up period was long, with a median of 5.3 years.

Some limitations of this study need to be recognized. First, this was a single-center, retrospective study, limiting the generalizability of the findings. Second, conservative treatment approaches were not uniform. Third, lack of photographs showing the healing process of the PA due to the retrospective nature. Fourth, risk factors of failure of conservative treatment were not identified because of the low proportion of patients undergoing surgery. Considering this limitation, the sample size needs to be expanded in the future. Fifth, the timing of surgery was not identified in this study, which will be addressed in our future studies. Last but not least, in our study, 80.2% of the universe of patients in the original series were excluded due to early surgical treatment, which resulted in a selection bias. Actually, the 153 patients were treated conservatively because their parents refused surgery. This was not due to the small abscess size. Indeed, the size of abscesses was large (≥ 1 cm) in 67 patients (43.8%). Moreover, we are currently conducting a prospective study of conservative treatment for PA in infants. In that study, the vast majority of patients are treated conservatively, which can reduce potential selection bias.

Despite these limitations, the present study has important clinical implications. The self-limiting nature will change the treatment paradigm of PA in infants. Before 2023, early surgical intervention was the main treatment for PA in infants at our hospital. Currently, nonoperative management is the first choice of treatment for PA in infants. We believe that our results will make more pediatric surgeons worldwide recognize the importance of nonoperative treatment. The avoidance of the expense of general anesthesia and surgical intervention are the advantages of nonoperative management. The paradigm shift in the management of PA in infants from an operative to a conservative approach undoubtedly has significant implications, especially in the era of minimally invasive surgery.

Despite these promising results, conservative treatment has not received much attention. Most pediatric surgeons still favor operative management^7,8,13,28–32^. In addition, many questions remain: (i) What drugs should be administered for the conservative treatment? (ii) Whether antibiotics should be used? (iii) What are the risk factors of failure of the conservative treatment? (iv) How to develop a predictive model for failure of the conservative treatment for PA in infants? (v) What’s the optimal timing of surgery? (vi) What should be the best surgical approach? More large-scale prospective studies will be necessary to address these questions.

## Conclusions

Conservative management seems to be promising and should be the first choice of treatment in most cases. Longer periods of conservative treatment may achieve better clinical outcomes in selected cases. Early surgical management of PA in infants should be avoided as much as possible. There will be a percentage of patients (about 10%) that would require surgical treatment.

## Data Availability

The datasets used and/or analyzed during the current study are available from the corresponding author upon reasonable request.

## Abbreviations

PA: Perianal abscess
ID: Incision and drainage
